# Supplementary material for: Hydroxychloroquine for prophylaxis of COVID-19 in health workers: A randomized clinical trial
Source: PLoS One. 2022 Feb 9;17(2):e0261980. doi: 10.1371/journal.pone.0261980 (PMC8827445; doi:10.1371/journal.pone.0261980)
Supplement: S1 Table — Multivariate models for the main variables that may be involved in hydroxychloroquine response and COVID-19 rate of infection. Regression was done with the Cox model, with days from recruitment to COVID-19 confirmed infection or end of study as the dependent variable. Multiple regression included all variables mentioned in the table. (PDF) [file pone.0261980.s001.pdf]

	Simple regression, unadjusted models			Multiple regression, adjusted models		
	Hazard ratio	95% confidence interval	p	Hazard ratio	95% confidence interval	p
Assigned intervention	0.18	0.021 - 1.48	0.11	0.18	0.20 - 1.56	0.12
Age at recruitment	0.94	0.85 - 1.05	0.27	0.94	0.83 - 1.05	0.26
Gender	1.00	0.22 - 4.45	0.99	1.41	0.26 - 7.58	0.69
Presence of concomitant disease	1.15	0.26 - 5.13	0.86	0.64	0.10 - 3.96	0.63
Body mass index	1.22	1.06 - 1.42	0.006	1.22	1.05 - 1.42	0.01
Profession (Nurse or physician/other)	2.13	0.41 - 10.9	0.37	1.93	0.36 - 10.33	0.44
Shift schedule (not night/night)	1.63	0.36 - 7.28	0.52	1.56	0.3 - 8.14	0.6
Days of exposition to COVID-19	0.99	0.96 - 1.03	0.73	1.00	0.96 - 1.04	0.96
