# Supplementary material for: Hydroxychloroquine for prophylaxis of COVID-19 in health workers: A randomized clinical trial
Source: PLoS One. 2022 Feb 9;17(2):e0261980. doi: 10.1371/journal.pone.0261980 (PMC8827445; doi:10.1371/journal.pone.0261980)
Supplement: S2 Table — Findings in laboratory studies and clinical examination throughout the study. P calculated with two-way ANOVA. H: subjects assigned to hydroxychloroquine intervention. P: subjects assigned to placebo intervention. Visit 1: recruitment Visit 2: after 30 days of treatment. Visit 3: after 60 days of treatment. BUN: blood urea nitrogen, ALT: alanine transferase, AST: aspartate transferase, RR: R to R interval in the electrocardiogram, QTc: corrected QT interval in the electrocardiogram, BMI: body mass index. (PDF) [file pone.0261980.s002.pdf]

	**Visit 1 (All) (n=130)**	**Visit 2 (All) (n=100)**	**Visit 3 (All n=80)**	**Visit 1 (P) (n=65)**	**Visit 1 (H) (n=65)**	**Visit 2 (P) (n=49)**	**Visit 2 (H) (n=51)**	**Visit 3 (P) (n=40)**	**Visit 3 (H) (n=40)**	p
**Leukocytes ($10^3/mm^3$)**	6.4 (5.5 - 7.9)	6.4 (5.6 - 7.3)	6.5 (5.75 - 7.41)	6.4 (5.4 - 8.1)	6.5 (5.5 - 7.4)	6.4 (5.6 - 7.4)	6.4 (5.6 - 7.1)	6.6 (5.75 - 7.6)	6.35 (5.75 - 7.25)	0.38
**Platelets ($10^3/mm^3$)**	248 (220 - 283)	250 (225 - 278)	257.5 (233 - 282.5)	248 (226 - 274)	248 (202 - 283)	254 (232 - 284)	244 (221 - 275)	253.5 (236 - 283.5)	259 (221.5 - 280)	0.24
**Lymphocytes ($10^3/mm^3$)**	2 (1.6 - 2.4)	2 (1.65 - 2.5)	1.95 (1.6 - 2.5)	2 (1.6 - 2.5)	2 (1.6 - 2.3)	2 (1.7 - 2.5)	2 (1.5 - 2.5)	1.95 (1.5 - 2.35)	1.95 (1.65 - 2.5)	0.45
**Neutrophils ($10^3/mm^3$)**	3.6 (3.1 - 4.6)	3.7 (3 - 4.65)	3.7 (3.1 - 4.8)	3.8 (3.1 - 4.7)	3.6 (2.9 - 4.5)	3.7 (3 - 4.7)	3.7 (3.1 - 4.5)	3.9 (3.2 - 4.8)	3.45 (3.05 - 4.75)	0.27
**Eosinophils ($10^3/mm^3$)**	0.1 (0.1 - 0.2)	0.1 (0.1 - 0.2)	0.1 (0.1 - 0.2)	0.1 (0.1 - 0.2)	0.1 (0.1 - 0.2)	0.1 (0.1 - 0.2)	0.1 (0.1 - 0.2)	0.1 (0.1 - 0.2)	0.1 (0.1 - 0.2)	0.51
**Creatinine (mg/dL)**	0.79 (0.67 - 0.94)	0.8 (0.692 - 0.915)	0.775 (0.67 - 0.915)	0.76 (0.65 - 0.88)	0.87 (0.68 - 0.95)	0.74 (0.66 - 0.89)	0.84 (0.71 - 0.92)	0.715 (0.655 - 0.815)	0.825 (0.72 - 0.925	0.83
**BUN (mg/dL)**	13 (11 - 15)	12 (11 - 15)	13 (10 - 15)	13 (11 - 15)	13 (11 - 15.17)	12 (11 - 15)	13 (11 - 16)	13.5 810 - 15)	13 (11 - 15.5)	0.91
**ALT**	19.5 (14 - 31)	18.5 (14 - 24)	18 (14 - 28)	18 (13 - 26)	21 (15 - 35.7)	18 (15 - 25)	20 (13 - 32)	18 (14 - 27.5)	18.5 (14 - 30)	0.3
**AST**	19 (16 - 24)	19 (17 - 24)	19 (16 - 23)	18 (15 - 22.3)	20 (18 - 27)	18 (16 - 22)	19 (17 - 25)	19 (16 - 22.5)	20 (16 - 23.5)	0.299
**Total bilirubin**	0.53 (0.41 - 0.79	0.54 (0.41 - 0.73)	0.52 (0.385 - 0.66)	0.53 (0.42 - 0.71)	0.53 (0.41 - 0.66)	00.52 (0.42 - 0.73)	0.57 (0.41 - 0.72)	0.455 (0.37 - 0.635)	0.525 0.395 - 0.74)	0.46
**Direct bilirubin**	0.1 (0.08 - 0.13)	0.1 (0.08 - 0.135)	0.1 (0.075 - 0.14)	0.1 (0.08 - 0.13)	0.1 (0.08 - 0.13)	0.1 (0.07 - 0.13)	0.11 (0.08 - 0.14)	0.09 (0.07 - 0.12)	0.12 (0.085 - 0.15)	0.28
**Systolic blood pressure (mmHg)**	112 (104 - 121)	111 (103 - 120)	110.5 (103.5 - 120)	112 (104 - 122)	112 (106 - 119)	111 (102 - 123)	112 (103 - 118)	110 (103 - 117.5)	113.5 (103.5 - 120	0.63
**Diastolic blood pressure (mmHg)**	71 (67 - 77)	70 (64 - 75)	71 (66.5 - 75)	72 (67 - 79)	71 (66 - 76)	69 (63 - 75)	72 (64 - 76)	72 (68 - 77.5)	70 (65 - 74.5)	0.049
**Heart rate (bpm)**	74 (67 - 81)	77 (70 - 85)	77.5 (69 - 84)	75 (67 - 81)	74 (66 - 78)	79 (70 - 86)	76 (69 - 82)	80 (71 86.5)	75 (66.5 - 81.5)	0.97
**Temperature (degrees Celsius)**	36.5 (36.3 - 36.8)	36.5 (36.3 - 36.7)	36.5 (36.4 - 36.7)	36.6 (36.3 - 36.7)	36.5 (36.3 - 36.8)	36.5 (36.4 - 36.7)	36.4 (36.3 - 36.7)	36.5 (36.4 - 36.7)	36.5 (36.4 - 36.7)	0.67
**RR**	22 (19 - 23)	22 (19 - 23)	20 (19 - 22)	22 (19 - 23)	22 (19 - 22)	22 (19 - 23)	22 (19 - 23)	21 (19 - 22)	20 (19 - 22)	0.43
**QTc**	407 (390 - 424)	409 (389 - 429)	412 (390 - 427)	414.5 (389 - 426.5)	403 (390 - 420	412 (386 - 433)	404 (389 - 428)	413.5 (381 - 427)	410 (394.5 424.5)	0.34
**Weight (kg)**	70.1 (60 - 80.7)	69 (60.2 - 78)	68.9 (60.7 - 77.65)	66.9 (59.9 - 80.8)	72.7 (60 - 80)	67.05 (60.2 - 76.8)	70 (60 - 80.3)	67.8 (61.2 - 76)	70.8 (60.45 - 79.3)	0.89
**Height (cm)**	161 (155 - 168)	161 (155 - 168)	160.5 (154 - 168.5)	159 (152 - 166)	163 (157 - 168)	159 (152 - 164)	163 (157 - 170)	157 (135 - 164)	163.5 (157 - 171)	0.052
**BMI (kg/m^2)**	26.8 (24.4 - 28.89)	26.4 (23.9 - 28.2)	26.1 (23.85 - 28.2)	26.85 (24.2 - 29.65)	26.2 (24.6 - 28.5)	26.65 (24.57 - 28.8)	26.1 (237 - 28.1)	26.5 (23.78 - 28.95)	25.95 (23.85 27.7	0.14
