# Supplementary material for: Hydroxychloroquine for prophylaxis of COVID-19 in health workers: A randomized clinical trial
Source: PLoS One. 2022 Feb 9;17(2):e0261980. doi: 10.1371/journal.pone.0261980 (PMC8827445; doi:10.1371/journal.pone.0261980)
Supplement: S3 Table — Adverse events by intervention, first and second follow up visit. Placebo: placebo group. HCQ: hydroxychloroquine group. P calculated with Fisher exact test, significative if lower than 0.05. (PDF) [file pone.0261980.s003.pdf]

	First follow up visit			Second follow up visit		
	Placebo	HCQ	p	Placebo	HCQ	p
Adverse effect at visit	20 (40%)	20 (39.22%)	0.896	25 (62.5%)	19 (47.5%)	0.157
Visual adverse effect	4 (8%)	1 (2%)	0.177	1 (2.5%)	0	0.58
Lack of focus	1	0		0	0	
Red spots	1	0		0	0	
Blurry vision	2	1		1	0	
Adverse cardiologic effects	3 (6%)	3 (5.88%)	0.664	1 (2.5%)	1 (2.5%)	0.672
Hypotension	1	2		0	0	
Thoracic opression	1	0		0	0	
Tachycardia	1	1		0	0	
Agitation	0	0		1	0	
Palpitations	0	0		0	1	
Adverse neurological effects	17 (34%)	11 (21.57%)	0.104	14 (35%)	9 (22.5%)	0.449
Agitation	0	0		1	0	
Dizziness	2	2		2	3	
Headache	14	8		13	8	
Paresthesia	4	1		1	1	
Lipothymia	0	1		0	0	
Adverse gastrointestinal effects	17 (34%)	24 (47.05%)	0.059	9 (22.5%)	8 (20%)	0.419
Vomit	0	1		1	0	
Nausea	5	4		2	3	
Diarrhea	9	16		7	4	
Epigastralgia	1	0		0	0	
Abdominal pain	1	2		0	1	
Gastritis	1	1		0	1	
Pyrosis	1	0		0	0	
Constipation	1	0		0	0	
Increased peristalsis	0	1		0	0	
Abdominal distension	0	2		1	0	
Hematochezia	0	1		0	0	
Colitis	0	0		1	0	
Adverse dermatologic effect	8 (16%)	3 (5.88%)	0.092	8 (20%)	1 (2.5%)	0.037

Pruritus	5	1		5	0	
Urticaria	2	1		0	1	
Hyperpigmentation	1	0		1	0	
Rash	0	1		0	0	
Alopecia	0	0		1	0	
Pustules	0	0		1	0	
Adverse audiological effect	3 (6%)	2 (3.92%)	0.5	1 (2.5%)	2 (5%)	0.382
Tinnitus	1	1		0	1	
Hearing loss	1	0		0	0	
Vertigo	2	0		1	0	
Right otalgia	0	1		0	1	
Other adverse effects	2 (4%)	3 (5.88%)	0.5	4 (10%)	3 (7.5%)	0.641
Arthralgia	2	0		1	0	
Night sweating	0	1		0	1	
Hyporexia	0	1		0	0	
Tiredness	0	1		0	1	
Odynophagia	0	0		1	0	
Rhinorrhea	0	0		1	0	
Abnormal transvaginal bleeding	0	0		0	1	
