# Supplementary material for: Hydroxychloroquine for prophylaxis of COVID-19 in health workers: A randomized clinical trial
Source: PLoS One. 2022 Feb 9;17(2):e0261980. doi: 10.1371/journal.pone.0261980 (PMC8827445; doi:10.1371/journal.pone.0261980)
Supplement: S1 File — (PDF) [file pone.0261980.s004.pdf]

TITULO DEL PROYECTO

Hidroxicloroquina para la profilaxis de COVID-19 sintomática en trabajadores de salud: Ensayo clínico aleatorizado.

INVESTIGADOR RESPONSABLE Y ADSCRIPCIÓN

Debe contener: nombre, título y adscripción del investigador principal

Dr. Jorge Rojas Serrano – Médico Adscrito al Servicio Clínico de Enfermedades Intersticiales

INVESTIGADORES PARTICIPANTES Y ADSCRIPCIONES

Debe contener: nombres, títulos y adscripciones de los investigadores participantes.

Dr. Cristóbal Guadarrama - jefe de la Unidad de Urgencias Respiratorias
Dra. Carmen Margarita Hernández Cárdenas - Jefe Áreas Críticas
Dr. Luis Felipe Jurado Camacho - Unidad de Cuidados Intensivos Respiratorios
Dr. José Rogelio Pérez Padilla - Departamento en Investigación de Tabaquismo y EPOC
Dra. Ireri Isadora Thirión Romero - Departamento en Investigación de Tabaquismo y EPOC
Dr. Sebastián Rodríguez Llamazares - Departamento en Investigación de Tabaquismo y EPOC
Dra. Alejandra Ramirez Venegas - Departamento en Investigación de Tabaquismo y EPOC

FECHA DE INICIO	FECHA DE TÉRMINO
23 Marzo 2020	22 Marzo 2021

CRONOGRAMA

Periodos en: **Meses:** X **Bimestres:** ____ **Otros** ____

	1	2	3	4	5	6	7	8	9	10	11	21	13	14	15	16	17	18	19	20	21	22	23	24
Ejecución	X	X	X	X	X																			
Análisis		x	x	x																				
Preparación de la publicación			x	x	x																			

ÚLTIMAS 5 PUBLICACIONES Y/O PROYECTOS DEL INVESTIGADOR RESPONSABLE

Instituto Nacional de Enfermedades Respiratorias Ismael Cosío Villegas INER
Comité de Investigación
25 MAR. 2020
APROBADO

Comité de Ética en Investigación
25 MAR. 2020
APROBADO
INER

1. Anti-HLA Class II Antibodies Correlate with C-Reactive Protein Levels in Patients with Rheumatoid Arthritis Associated with Interstitial Lung Disease. Del Angel-Pablo AD, Buendía-Roldán I, Mejía M, Pérez-Rubio G, Nava-Quiroz KJ, **Rojas-Serrano J**, Falfán-Valencia R. Cells. 2020 Mar 11;9(3):E691. doi: 10.3390/cells9030691.PMID: 32168865
2. An Open-label Study With Pirfenidone on Chronic Hypersensitivity Pneumonitis. Mateos-Toledo H, Mejía-Ávila M, Rodríguez-Barreto Ó, Mejía-Hurtado JG, Rojas-Serrano J, Estrada A, Castillo-Pedroza J, Castillo-Castillo K, Gaxiola M, Buendía-Roldan I, Selman M.Arch Bronconeumol. 2020 Mar;56(3):163-169. doi: 10.1016/j.arbres.2019.08.019. Epub 2019 Nov 26.PMID: 31784348 English, Spanish.
3. Influence of Antisynthetase Antibodies Specificities on Antisynthetase Syndrome Clinical Spectrum Time Course. Cavagna L, Trallero-Araguás E, Meloni F, Cavazzana I, Rojas-Serrano J, et. Al..J Clin Med. 2019 Nov 18;8(11):2013. doi: 10.3390/jcm8112013.PMID: 31752231
4. Quantitative assessment of interstitial lung disease in Sjögren's syndrome.Guisado-Vasco P, Silva M, Duarte-Millán MA, Sambataro G, et al. Rojas-Serrano J, Vanchieri C .PLoS One. 2019 Nov 8;14(11):e0224772. doi: 10.1371/journal.pone.0224772. eCollection 2019.PMID: 31703067
5. The immune response to Hymenolepis nana in mice decreases tumorigenesis induced by 7,12 dimethylbenz-anthracene. Ramos-Martínez E, Rojas-Serrano J, García-Hernández O, García-Vázquez FJ, Andrade WA, Avila G, Salinas-Pasquier L, López-Vancell MR.Cytokine. 2019 Nov;123:154743. doi: 10.1016/j.cyto.2019.154743. Epub 2019 Jun 27.PMID: 31255915

TITULO DEL PROYECTO

Hidroxicloroquina para la profilaxis de COVID-19 sintomática en trabajadores de salud: Ensayo clínico aleatorizado.

DEFINICIÓN DEL PROBLEMA

El personal de salud, tiene alto riesgo de infección por COVID-19 (1). En China, hasta principios de marzo, se han reportado más de 3300 infecciones por COVID-19 en el personal médico y en algunos hospitales como el Clinic de Barcelona, el 23% de los médicos han contraído la infección por COVID19. En Italia, se ha estimado la incidencia de infección por COVID-19 en una cifra muy similar de personal médico infectado con COVID19 de un 20% (1%). Otro problema grave, es que el personal sanitario puede llevar la infección por COVID19 a su hogar, ya se ha reportado transmisión a miembros de familia de estos trabajadores, además, el personal médico puede ser un vector tanto intrahospitalario, llegando a diseminar la infección a pacientes atendidos por otra causa, o bien, al resto del equipo médico.

La enfermedad en el personal de salud es un problema muy grave en emergencias sanitarias, ya que limita la posibilidad de atención, debilita al personal y reduce su motivación. De hecho, en China, se movilizaron 40,000 médicos de otras regiones para apoyar a Wuhan y a la provincia en el brote inicial. También en Italia, apareció una noticia de que consideraban contratar médicos de otros países incluyendo médicos de Cuba, China y Venezuela para apoyar en la atención médica de los pacientes afectados, debido a que los sistemas de salud de Italia están totalmente rebasados.

Instituto Nacional de Enfermedades Respiratorias Ismael Cosío Villegas
Comité de Investigación
25 MAR. 2020
APROBADO
INER

Instituto Nacional de Enfermedades Respiratorias Ismael Cosío Villegas
Comité de Ética en Investigación
25 MAR. 2020
APROBADO
INER

PREGUNTA DE INVESTIGACIÓN

¿El tratamiento profiláctico con Hidroxicloroquina 200 mg/día por 60 días reduce significativamente la incidencia de infecciones sintomáticas por COVID-19, en el personal de salud en contacto con pacientes infectados comparado contra placebo?

25 MAR. 2020 APROBADO INER

25 MAR. 2020 APROBADO INER

ANTECEDENTES

Se deberá proveer información de base para poder entender la relevancia del presente estudio y el propósito y fundamento del estudio.

Se deberá incluir un resumen de los datos de estudios no clínicos que tengan potencialmente una importancia clínica y de estudios clínicos y epidemiológicos relevantes para el presente estudio, así como las referencias de la literatura relevantes al estudio y que provean un sustento para el estudio.

El brote de infección respiratoria por el coronavirus 2019 (COVID-19) inició en China, en Diciembre del 2019, en la ciudad de Wuhan, provincia de Hubei [1-5]. De esta ciudad, el brote se ha ido extendiendo a varias provincias de China y a otros países [6]. Hasta el 16 de Febrero del 2020, se habían reconocido 28 países con casos confirmados, con 70,548 infecciones y 1770 muertes en China, y 413 infecciones en Japón [6]. Como en otros coronavirus, el del SARS, y el del MERS, la infección original humana probablemente se obtuvo de contacto con animales, pero se ha demostrado una eficaz transmisión entre humanos, con una tasa de crecimiento mayor a 1, estimada de 2.6 [7], y una duplicación rápida de casos que aseguran no solo el mantenimiento de la epidemia si no su expansión, tal y como se ha observado.

La gama de presentaciones clínicas del COVID-19, es amplia, desde infecciones asintomáticas hasta infecciones fatales, con daño pulmonar grave, falla respiratoria, y falla multiorgánica. El daño grave se ha asociado, como en la influenza grave, a una reacción inflamatoria sistémica o una tormenta de citocinas, que podría ser atenuada por el efecto inmunomodulador de la cloroquina o hidroxicloroquina. La tasa de letalidad general de la infección por COVID-19 se ha estimado alrededor del 2%, pero en los casos graves en falla respiratoria, o requiriendo cuidados críticos, es considerablemente mayor, entre 10-15%, pero fácilmente podría incrementarse hasta el 20-30% dependiendo de los criterios de entrada a una unidad de terapia intensiva.

Hasta el momento no se cuenta con un tratamiento efectivo disponible para el COVID-19, sin embargo, in vitro, se reportó recientemente la efectividad in vitro del Remdesivir y de la cloroquina. Esta última, en concentraciones capaces de ser obtenidas in vivo [8]. La cloroquina e hidroxicloroquina (HCQ) son medicamentos originalmente antipalúdicos, químicamente casi idénticos, en uso regular por décadas y con un perfil de seguridad adecuado y un costo accesible. La principal toxicidad de la HCQ, fármaco en investigación en este ensayo, es la retinopatía, los factores de riesgo asociados a la misma son dosis mayores a 5 mg/kg peso/día, el uso prolongado (> 10 años) y el antecedente de insuficiencia renal, así como el uso de tamoxifen. Otros efectos adversos reportados con la HCQ, que ocurren de manera muy poco frecuente, son la presencia de alteraciones de la conducción cardiaca, cardiomiopatías, síntomas neurológicos como nistagmus, cefalea, insomnio, pesadillas, entre otros.

El efecto antiviral de la HCQ, se ha atribuido al incremento del pH de los endosomas, que se requieren para la fusión del virus con la célula y también por interferencia con la glicosilación de los receptores celulares del coronavirus en cuestión (SARS-cov-2) [9-11]. Además del efecto antiviral, la HCQ tiene efectos inmunomoduladores, por lo que es usado también para el tratamiento de enfermedades como el Lupus Eritematoso Sistémico y la Artritis Reumatoide. Adicionalmente, el efecto antinflamatorio también podría potenciar el efecto antiviral y el impacto sistémico de las infecciones graves. En caso de ser efectivo omo profilaxis, podría ser un medicamento de gran utilidad en disminuir el riesgo de infección por COVId 19 en el personal de salud.

Recientemente se publicó una carta[12] que describe que en Febrero 17, el Consejo Estatal de China dio un aviso de prensa indicando que la cloroquina había demostrado marcada eficacia y seguridad aceptable para el tratamiento de la neumonía por COVID-19 en ensayos multicéntricos de China con más de 100 pacientes de

10 hospitales. Por otra parte, han salido reportes de éxito terapéutico utilizando hidroxicloroquina en combianción con azitromicina, todos estos estudios son preliminares, sin embargo apoyan la tesis del efecto antiviral de la HCQ y su posible uso como profilaxis en sujetos en riesgo de infección por COVID-19. Con base en lo anterior, se propone este estudio clínico, doble ciego, con distribución al azar, de dos grupos paralelos, de tratamiento con HCQ contra placebo, para evaluar la eficacia y seguridad de HCQ en el personal de salud con riesgo de contraer infección por COVID-19,

JUSTIFICACIÓN

Se carece de un tratamiento farmacológico aprobado para la quimioprofilaxis contra COVID-19 y, basados en la efectividad in-vitro de la HCQ contra el virus, el perfil de seguridad de la HCQ y su bajo costo, se propone un estudio clínico controlado, con distribución al azar, doble ciego, para evaluar la eficacia y seguridad de la HCQ para prevenir infecciones sintomáticas y graves en el personal de la salud altamente expuesto a infección por COVID-19. La dosis propuesta para profilaxis es de 200 mg/día. Esta dosis es menor a 5 mg/kg de peso día en sujetos ≥ 50 kg de peso, por lo que el riesgo de toxicidad retiniana es mínimo, ya que los sujetos < 50 kg serán excluidos. En caso de demostrar que la incidencia de infecciones sintomáticas y graves de COVID 19 en el personal de salud disminuyen significativamente, este estrategia de profilaxis puede ser una de las mejores herramientas para mantener la seguridad del personal de salud expuesto a COVID-19

HIPÓTESIS

Ha: El tratamiento profiláctico con Hidroxicloroquina 200 mg/día por 60 días reduce la incidencia de infecciones sintomáticas (> 10%) por el SARScov-2 en el personal de salud en contacto con pacientes infectados.

Ho: El tratamiento profiláctico con Hidroxicloroquina 200 mg/día por 60 días no reduce la incidencia de infecciones sintomáticas por el SARScov-2 en el personal de salud en contacto con pacientes infectados.

Instituto Nacional de Enfermedades Respiratorias Ismael Cosío Villegas INER
Comité de Investigación
25 MAR. 2020
APROBADO

Instituto Nacional de Enfermedades Respiratorias Ismael Cosío Villegas INER
Comité de Ética en Investigación
25 MAR. 2020
APROBADO

OBJETIVO (S) GENERAL (ES)

Evaluar la eficacia de la profilaxis con HCQ a una dosis de 200 mg día, comparada contra placebo para disminuir la incidencia de infección sintomática por COVID-19 en trabajadores de la salud con alta exposición a COVID-19

Evaluar la seguridad de la profilaxis con HCQ a una dosis de 200 mg día, comparada contra placebo, en la profilaxis para disminuir la incidencia de infección sintomática por COVID-19 en trabajadores de la salud con alta exposición a COVID-19.

OBJETIVOS ESPECÍFICOS

1. Comparar el riesgo de infección sintomática por COVID19 en sujetos tratados con profilaxis con hidroxicloroquina contra el riesgo de infección sintomática por COVID 19 en sujetos tratados con placebo.
2. Estimar el riesgo de sufrir el desenlace compuesto formado por: ingreso a terapia intensiva, necesidad de ventilación mecánica no invasiva/ invasiva y muerte, entre los sujetos con alta exposición a COVID19, tratados con profilaxis de HQC contra los tratados con placebo.
3. Estimar el riesgo de eventos adversos en personal de la salud tratado con profilaxis con hidroxicloroquina contra el riesgo de eventos adversos en los sujetos tratados con placebo.

DISEÑO EXPERIMENTAL

1. INVESTIGACIÓN CLÍNICA X
2. INVESTIGACIÓN BÁSICA
3. INVESTIGACIÓN SOCIAL
4. INVESTIGACIÓN EPIDEMIOLÓGICA

FAVOR DE INDICAR EL TIPO DE ESTUDIO

	OBSERVACIONAL
X	EXPERIMENTAL
	RETROSPECTIVO
X	LONGITUDINAL
X	PROSPECTIVO
	TRANSVERSAL
	OTRO FAVOR DE INDICAR:

"Indicar lo que se pretende obtener para el conocimiento en el tema de estudio al desarrollar esta investigación"

1. Comprensión
2. Prevención X
3. Diagnóstico
4. Tratamiento
5. Rehabilitación

Indicar la línea de investigación de la que se deriva este proyecto:

1. Programa de Estudio sobre la Influenza y otros Virus Respiratorios. X
2. Programa de Estudio sobre Problemas Respiratorios asociados a la exposición al Tabaco y Humo de Leña.
3. Programa de Estudio sobre Tuberculosis.
4. Programa de Estudio sobre Enfermedades Fibrosantes del Pulmón.
5. Programa de Estudio sobre Cáncer Pulmonar.
6. Programa de Estudio sobre Asma.
7. Programa de Estudio de la Clínica del Sueño.
8. Programa de Estudio sobre VIH y SIDA.
9. Programa de Estudio en Cirugía y Trasplante Pulmonar Experimental.
10. Programa de Estudio de Investigación en Inmunogenética y Alergia.
11. Programa de Estudio sobre Infecciones Respiratorias Agudas.
12. Programa de Estudio sobre Enfermedades del Oído y vías Respiratorias Altas.
13. Programa de Estudio sobre Enfermedades Reumáticas.
14. Programa de Estudio sobre Enfermedades Metabólicas asociadas a Enfermedades Respiratorias.
15. Programa de Estudio sobre Fisiología Respiratoria.
16. Programa de Estudio sobre Tromboembolia Pulmonar.
17. Programa de Estudio sobre Enfermedades Cardiovasculares y Respiratorias.
18. Programa de Investigación sobre Salud Respiratoria.
19. Programa de Estudio sobre Farmacología.
20. Programa de Desarrollo Biotecnológico.
21. Otro
    Especificar:

Instituto Nacional de Enfermedades Respiratorias Ismael Cosío Villegas
Comité de Investigación
25 MAR. 2020
APROBADO
INER

Instituto Nacional de Enfermedades Respiratorias Ismael Cosío Villegas
Comité de Ética en Investigación
25 MAR. 2020
APROBADO
INER

MATERIAL Y MÉTODOS

UTILIZAR LAS HOJAS NECESARIAS

A) Lugar del estudio
B) Descripción de la población de estudio
C) Procedimientos del estudio
D) Número necesario de sujetos de investigación
E) Criterios de inclusión y exclusión
F) Captura, procesamiento, análisis e interpretación de la información.

A) **LUGAR DEL ESTUDIO:** Instituto Nacional de Enfermedades Respiratorias: áreas de los servicios de urgencias, medicina crítica y servicios clínicos de hospitalización

B) **DESCRIPCION DE LA POBLACIÓN:** Personal de salud del INER en contacto con la atención de pacientes con COVID-19: incluye personal médico, enfermería, y paramédicos como camilleros, y personal de limpieza. El personal del INER que será incluido, ha tomado cursos de capacitación para utilizar equipo de protección de bioseguridad, con el fin de disminuir el riesgo de infección por COVID-19.

C) **PROCEDIMIENTOS DEL ESTUDIO:** Ensayo clínico controlado, fase III, doble ciego, con grupos paralelos con distribución al azar.

Aleatorización: Se distribuirá al azar, de forma estratificada a trabajadores de salud expuestos a pacientes con diagnóstico de COVID–19. Los estratos para la aleatorización serán los siguientes:

A) **Grupo A:** Sujetos con exposición de alto riesgo a COVID-19; definidos como aquel personal que realiza invasivos de alto riesgo (broncoscopías, intubación, extubación y/o toma de muestras respiratorias), cuidados de paciente crítico o bajo ventilación mecánica.

B) **Grupo B:** Sujetos con exposición de moderado a bajo a COVID–19; definida como aquel personal que realiza atención a pacientes en áreas de triage, consulta externa o cuya participación en el servicio clínico corresponde a labores administrativas o de intendencia.

El procedimiento de distribución al azar se realizará con el método de bloques permutados con distribución al azar, en bloques de cuatro pares

Descripción del ciego: Participante, tratante, investigador, monitor de desenlaces, análisis de resultados.

Desenlace primario: infección sintomática (Tos seca, con o sin disnea, con o sin fiebre) por COVID 19, demostrada mediante PCR-TR de acuerdo con el protocolo de la OMS.

Desenlace secundarios:

1. Ingreso a la unidad de terapia intensiva, necesidad de ventilación mecánica invasiva/no invasiva, muerte, secundaria a infección por COVID-19, demostrada por PCR-TR de acuerdo con el protocolo de la OMS..
2. Incidencia y gravedad de eventos adversos.

   Evento adverso: todo evento médico en los sujetos incluidos en el ensayo. El evento adverso no necesariamente debe tener una relación causal con le tratamiento estudiado: ejemplos de eventos adversos incluyen síntomas y signos anormales, reacciones de hipersensibilidad, o alteraciones de las pruebas de laboratorio.

   Evento adverso grave: se define como un evento que resulta en la muerte del sujeto, en un evento que comprometa la vida, que requiera hospitalización o resulte en incapacidad.

   Debido a los efectos ya conocidos de la HCQ se tomará en especial atención la incidencia de los siguientes eventos adversos:

Comité de Investigación
25 MAR. 2020
APROBADO

Comité de Ética en Investigación
25 MAR. 2020
APROBADO
INER

a) Reacción cutánea, generalmente al utilizarse en conjunto con otros fármacos.
b) Supresión medular, misma que deberá vigilarse mensualmente a través de biometría hemática de control.
c) Hipoglucemia grave en cuyo caso debe suspenderse el tratamiento.
d) Miopatía proximal con debilidad proximal o atrofia muscular.
e) Aumento de riesgo suicida de acuerdo con la escala C-ssrs

Se define como reacción adversa a la respuesta ante un compuesto que es nocivo e involuntario, que ocurre a dosis que normalmente son administradas a seres humanos. Las reacciones adversas descritas son:

1-10% oftalmológicas, dependiente de la dosis

Frecuencia no definida: dermatológicas; endocrinas; metabólicas; gastrointestinales; hematológicas; hepáticas; sistema nervioso central; inmunológicas.

Graduación de las reacciones adversas:

Grado 1: evento adverso leve; se refiere a los efectos que pudieran suceder por el compuesto que son temporales y no causan daño permanente o incapacidad.

Grado 2: evento adverso moderado; se refiere a los efectos que pudieran suceder por el compuesto que son temporales pero requieren el apoyo de otras terapéuticas para poder corregirse y no causan daño permanente o incapacidad.

Grado 3: evento adverso grave; se refiere a los efectos que pudieran suceder por el compuesto que pueden ser temporales o permanentes, sin riesgo de mortalidad o de discapacidad.

Grado 4: evento adverso con riesgo de mortalidad o de discapacidad

Valorado por los siguientes estudios:

ECG: prolongación del intervalo QT.

Biometría hemática: supresión de médula ósea, definida con los siguientes valores: neutrófilos <500/microL plaquetas <20,000/microL; reticulocitos <20,000/microL.
Insuficiencia hepática: INR >1.4, ALT > 33 units/L, AST > 55, bilirrubinas totales>4 mg/dL, cuenta plaquetaria $\leq$150,000/mm^3

Debido a la naturaleza del estudio, que es una población cautiva que acude al INER al menos cinco veces a la semana, es posible estimar los días al desenlace, se les instruirá a los participantes del estudio, a informar inmediatamente a los investigadores del estudio, en caso de presentar tos, fiebre, disnea, o algún otro síntoma o signo de nueva aparición. De manera semanal, los investigadores del estudio preguntarán a los sujetos incluidos acerca de síntomas asociados a la infección por COVID-19, así como posibles eventos adversos.

Si algún sujeto del protocolo presentará reacciones adversas se iniciaría el tratamiento inmediato, así como su notificación. El tratamiento inicial se llevaría acabo en el INER, si es necesario se solicitaría el apoyo de otras Instituciones, sin costo para el sujeto.

Grupo experimental: Recibe hidroxicloroquina por vía oral, 200 mg al día por 60 días. De acuerdo con el resultado del análisis interim a las 4 semanas y 8 semanas, o el final, se decidirá quitar o no el ciego y dar o no profilaxis a los dos grupos. De acuerdo con los resultados del ensayo, se decidirá hacer una enmienda para continuar con una fase de seguimiento del protocolo abierta, con profilaxis con HCQ.

Grupo control: Placebo similar por 60 días.

Velocidad de reclutamiento: Se incluirían el total del personal asignado conforme se llegue a sus turnos de trabajo.

Fecha de inicio de reclutamiento: Inmediatamente posterior al proceso de revisión y en su caso, aprobación del protocolo,

Fecha de terminación:

Pruebas para realizar:

- Pruebas mínimas al ingreso (previo a la aleatorización) y a los 30 y 60 días.
    - Biometría hemática
    - Electrolitos Séricos
    - Química Sanguínea de 3 elementos: para evaluar el efecto en los niveles de glucosa en sangre, y descartar de manera basal DM.
    - Perfil Hepático
    - Electrocardiograma: para evaluar la incidencia de alteraciones en la conducción cardiaca
- Panel de 10 virus respiratorios y de SARSCoV-2 sólo en caso de tener síntomas

A los 30 y 60 días: Vigilancia de eventos adversos.

D) **NÚMERO NECESARIO DE SUJETOS:** **Nivel de significancia**: Alfa 5% a 2 colas. **Potencia:** (1-beta, posibilidad de detección): 90% **Cálculo final:** De acuerdo con el análisis estadístico propuesto, la diferencia en el tiempo al desenlace se realizará con una prueba de Log Rank, utilizando las siguiente fórmulas:

$$\boldsymbol{n} = \frac{\boldsymbol{d}}{2-S1(\infty)-S2(\infty)} \text{ y } d = (Z\alpha(2) - Z1 - \beta)^2$$

Tomando en cuenta una diferencia de 10% de mayor supervivencia libre de infección pro COVID-19 y tomando en cuenta una supervencia reportada libre de infección por COVID19 de 76%, se obtiene un tamaño de muestra de 159 por grupo, tomando en cuenta las pérdidas, se decide incluir a 200 sujetos por grupo, para un total de 400 sujetos en total

CRITERIOS DE INCLUSIÓN Y EXCLUSIÓN

Criterios de inclusión al estudio:

- Al menos 18 años cumplidos.
- Ser trabajador de salud expuesto a pacientes con enfermedad respiratoria por COVID-19: personal médico, químico, de enfermería, intendencia, camillería y transporte, administrativo y terapia respiratoria, con entrenamiento en medidas de bioseguridad, y adecuado uso de equipo de protección para disminuir el riesgo de infección por COVID-19.
- Ausencia de síntoma respiratorios al momento de la aleatorización e inicio de tratamiento/placebo.
- Consentimiento informado, que incluye la aleatorización a cualquier brazo.

Comité de Investigación 25 MAR. 2020 APROBADO INER

Criterios de exclusión:

- Hipersensibilidad conocida a hidroxicloroquina manifestada por anafilaxia.
- Consumo actual de hidroxicloroquina o cloroquina
- Peso menor a < 50 kg al momento de la evaluación basal.
- Mujer con fecha de última menstruación mayor a un mes que no cuente con prueba de embarazo.
- Mujer con prueba de embarazo positiva.
- Mujer en proceso de lactancia.
- Antecedente de enfermedad hepática crónica. (Child-Pugh B o C).
- Antecedente de enfermedad renal crónica. (TFG menor o igual a 30).
- Antecedente de uso de tamoxifeno.
- Antecedente de retinopatía por cualquier causa, y/o síntomas disminución de visión central, disminución de agudeza visual nocturna, visión borrosa, afección de la visión a colores.

Criterios de eliminación

- Decisión del participante o representante legal de retirarse del estudio.

Comité de Investigación 25 MAR. 2020 APROBADO INER

E) ANALISIS ESTADÍSTICO

Las variables categóricas se describirán con frecuencias y porcentajes; las numéricas con media y DE, o medianas e intervalos intercuartilartes de acuerdo a su distribución. Se estimará el tiempo al desenlace de infección por COVID-19, el tiempo al desenlace compuesto, tanto de los sujetos del grupo experimental como del placebo. Se estimará la función de supervivencia con el método de Kaplan-Meier y la prueba de hipótesis para comparar el tiempo al desenlace de los grupos de estudio se hará con la prueba de Log Rank. El Hazard ratio y su intervalo de confianza al 95% se usará como medida de fuerza de asociación para evaluar el efecto de la dosis profilactica de HCQ sobre el desenlace primario y el secundario del estudio. Se estimará el efecto de la profilaxis en los dos estratos propuestos en el estudio, tanto con la prueba de Log Rank y se evaluarqa la interacción entre la profilaxis y el estrato con el método de regresión de Cox. Debido a la urgencia de conocer los resultados del estudio, se planean los siguientes análisis interim: al momento de que el 50% de la muestra cumpla 4 semanas de seguimiento, y a las 8 semanas de que el 50% de la muestra tenga ese seguimiento. Los sujetos que no presenten el desenlace serán censurados en su seguimiento a los 3 meses desde la distribución al azar y el inicio del tratamiento/placebo. Se propone un análisis multivariado de riesgos proporcionales de Cox, para evaluar los riesgos asociados a la infección por COVID19, y riesgo del desenlace compuesto. Todos los análisis son a dos colas, la estrategia de análisis es por intención a tratar, así que todo sujeto sometido al procedimiento de distribución al azar será incluido en el análisis.

Comité de monitorización de datos y seguridad (CMDS, en inglés DSMC)

Se contará con un CMDS, grupo multidisciplinario asesor, formado por un experto en estadística, un investigador clínico, un experto en ética y un clínico relacionado con la influenza y coronavirus ninguno involucrado en el ensayo clínico y por lo tanto independientes, quienes, a los tiempos propuestos, tendrán acceso a los datos y al código de aleatorización para que evalúen los datos incluyendo la seguridad y los resultados.

Investigador Clínico: Gustavo Lugo, farmacólogo clínico, anestesiólogo e intensivista quien actuara como coordinador.

Estadística: Dra. Rosario Fernández, Jefe del Departamento de epidemiología del INER.

Etica: Dr. Patricio Santillán, director médico

Clínico: Dr. Arturo Martínez, Infectólogo, experto en las infecciones tipo influenza y ahora en la infección por COVID-19

Su objetivo es valorar ventajas y desventajas de continuar el ensayo en el análisis a la mitad de la muestra, sobre todo si se considera ético continuar el reclutamiento, dados los resultados encontrados a la mitad del estudio. En principio, no se cuenta con estudios controlados con qué comparar pero pueden aparecer en el transcurso del ensayo.

Se planea un análisis interino, al completar la mitad de la muestra, que pueda identificar si se encuentra una diferencia notoria en la medida de resultado principal, que demuestre una ventaja en efectividad, que por su magnitud orille a parar el ensayo. También para indicar efectos adversos significativos o toxicidad. El análisis estadístico final, se realizará tomando en cuenta este análisis intermedio.

2 5 [illegible]
2 5 [illegible]
INER

Comité de Ética en Investigación
2 5 MAR. 2020
APROBADO
INER

CONSIDERACIONES ÉTICAS

a) Indique el tipo de riesgo de la investigación.

El tipo de riesgo deberá ser considerado de a cuerdo a lo establecido en el Art. 17 del REGLAMENTO de la Ley General de Salud en Materia de Investigación para la Salud:

- Investigación sin riesgo.
- Investigación con riesgo mínimo.
- Investigación con riesgo mayor que el mínimo.

Comité de Investigación
2 5 MAR. 2020
APROBADO
INER

b) Para los protocolos de investigación en animales, hacer mención de la reglamentación vigente:

- Reglamento de la Ley General de Salud en Materia de Investigación para la Salud, Título Séptimo, de la Investigación que incluya la utilización de animales de experimentación.
- Ley Federal de Sanidad Animal.
- Reglamento de la Ley Federal de Sanidad Animal.
- NOM-062-ZOO-1999, Especificaciones técnicas para la producción, cuidado y uso de animales de laboratorio.
- Guía para el cuidado y uso de los animales de laboratorio "Guide for the Care and use of Laboratory Animals eigth Edition, Institute for Laboratory Animal Research Council".
- Lineamientos para el sacrificio humanitario de los animales "AVMA Guidelines for the Euthanasia of Animals: 2013 Edition".

INVESTIGACIÓN CON RIESGO MAYOR AL MÍNIMO

Todo sujeto será incluido únicamente previa conducción del consentimiento informado.
Los riesgos de los sujetos que decidan participar en el estudio son los siguientes:

1. Por la toma de muestra de sangre basal, a los 30 y 60 días, los riesgos asociados son dolor a la venopunción, riesgo de infección y formación de hematoma en el sitio de la punción.
2. Los risgos asociados a la toma de electrocsrdiograma, son la presencia de equimosis por los chupones del equipo.
3. Los riesgos asociados a la toma de muestra por hisopado, nasal o faríngeo, para el PCR-TR de detección del COVID-19 son la presencia de dolor, erosión leve de la mucosa faríngea con o sin sangrado leve.

La expectativa de los efectos del tratamiento con hidroxicloroquina en caso de ser asignado al brazo de tratamiento es que exista una disminución en el riesgo de padecer infección sintomática por COVID-19 pero con un mayor riesgo de presentar efectos adversos.de la profilaxis
La expectativa de los efectos del placebo en caso de ser asignado a dicho brazo es mantener un riesgo de padecer infección sintomática por COVID-19 que, si no hubiera participado en el estudio, pero a expensas de presentar las molestias previamente mencionadas relacionadas con la toma de muestras.
En caso de que el ensayo demuestre un claro efecto benéfico de la profilaxis con HCQ en la disminución de la incidencia de infección por COVID-19, se propondrá una enmienda al protocolo, para quitar el ciego, ya sea en la semana 4, en la semana 8 o al final de los 3 meses de seguimiento originalmente propuesto.

COMENTARIOS Y CONSIDERACIONES DE LOS INVESTIGADORES
FORMACIÓN DE NUEVOS RECURSOS HUMANOS DURANTE EL PROYECTO
(INDICAR NIVEL)

El protocolo forma parte de la tesis para obtener el grado de Doctor en Ciencias del alumno Luis Felipe Jurado Camachoj

2 5 [illegible]
2 5 [illegible] 2020
APROBADO
INER

SIN TEXTO

Comité de Investigación
25 MAR. 2020
APROBADO
INER

Comité de Ética en Investigación
25 MAR. 2020
APROBADO
INER

REFERENCIAS BIBLIOGRÁFICAS

UTILIZAR LAS HOJAS NECESARIAS

1. Joob B, Wiwanitkit V. COVID-19 in medical personnel: observation from Thailand. *The Journal of hospital infection* 2020.
2. Chan JF, Yuan S, Kok KH, To KK, Chu H, Yang J, Xing F, Liu J, Yip CC, Poon RW, Tsoi HW, Lo SK, Chan KH, Poon VK, Chan WM, Ip JD, Cai JP, Cheng VC, Chen H, Hui CK, Yuen KY. A familial cluster of pneumonia associated with the 2019 novel coronavirus indicating person-to-person transmission: a study of a family cluster. *Lancet* 2020.
3. Chen N, Zhou M, Dong X, Qu J, Gong F, Han Y, Qiu Y, Wang J, Liu Y, Wei Y, Xia J, Yu T, Zhang X, Zhang L. Epidemiological and clinical characteristics of 99 cases of 2019 novel coronavirus pneumonia in Wuhan, China: a descriptive study. *Lancet* 2020.
4. Holshue ML, DeBolt C, Lindquist S, Lofy KH, Wiesman J, Bruce H, Spitters C, Ericson K, Wilkerson S, Tural A, Diaz G, Cohn A, Fox L, Patel A, Gerber SI, Kim L, Tong S, Lu X, Lindstrom S, Pallansch MA, Weldon WC, Biggs HM, Uyeki TM, Pillai SK, Washington State -nCo VCIT. First Case of 2019 Novel Coronavirus in the United States. *The New England journal of medicine* 2020.
5. Zhou P, Yang XL, Wang XG, Hu B, Zhang L, Zhang W, Si HR, Zhu Y, Li B, Huang CL, Chen HD, Chen J, Luo Y, Guo H, Jiang RD, Liu MQ, Chen Y, Shen XR, Wang X, Zheng XS, Zhao K, Chen QJ, Deng F, Liu LL, Yan B, Zhan FX, Wang YY, Xiao GF, Shi ZL. A pneumonia outbreak associated with a new coronavirus of probable bat origin. *Nature* 2020.
6. Zhu N, Zhang D, Wang W, Li X, Yang B, Song J, Zhao X, Huang B, Shi W, Lu R, Niu P, Zhan F, Ma X, Wang D, Xu W, Wu G, Gao GF, Tan W, China Novel Coronavirus I, Research T. A Novel Coronavirus from Patients with Pneumonia in China, 2019. *The New England journal of medicine* 2020.
7. Centers of Disease Control. 2019 Novel Coronavirus. 2020 [cited 2020 February 7]; Available from: https://www.cdc.gov/coronavirus/2019-ncov/locations-confirmed-cases.html
8. Zhao S, Musa SS, Lin Q, Ran J, Yang G, Wang W, Lou Y, Yang L, Gao D, He D, Wang MH. Estimating the Unreported Number of Novel Coronavirus (2019-nCoV) Cases in China in the First Half of January 2020: A Data-Driven Modelling Analysis of the Early Outbreak. *Journal of clinical medicine* 2020: 9(2).
9. Wang M, Cao R, Zhang L, Yang X, Liu J, Xu M, Shi Z, Hu Z, Zhong W, Xiao G. Remdesivir and chloroquine effectively inhibit the recently emerged novel coronavirus (2019-nCoV) in vitro. *Cell research* 2020.
10. Savarino A. Use of chloroquine in viral diseases. *The Lancet Infectious diseases* 2011: 11(9): 653-654.
11. Savarino A, Boelaert JR, Cassone A, Majori G, Cauda R. Effects of chloroquine on viral infections: an old drug against today's diseases? *The Lancet Infectious diseases* 2003: 3(11): 722-727.
12. Yan Y, Zou Z, Sun Y, Li X, Xu KF, Wei Y, Jin N, Jiang C. Anti-malaria drug chloroquine is highly effective in treating avian influenza A H5N1 virus infection in an animal model. *Cell research* 2013: 23(2): 300-302.
13. Gao J, Tian Z, Yang X. Breakthrough: Chloroquine phosphate has shown apparent efficacy in treatment of COVID-19 associated pneumonia in clinical studies. *Bioscience trends* 2020.

DESGLOSE DE RECURSOS HUMANOS Y TÉCNICOS REQUERIDOS PARA EL ESTUDIO

Especificar aquellos que requieran ser autorizados por la Subdirección de Servicios Auxiliares de Diagnóstico y Paramédicos

RECURSO	NÚMERO REQUERIDO	ORIGEN O SITIO DONDE SE REALIZA	FUENTE DEL FINANCIAMIENTO
Investigador principal	1	INER	INER
Sub investigadores	8	INER	INER
Coordinador de estudio	1	INER	CONACYT
Ejecutadores	3	INER	CONACYT
Kits de laboratorio	400	INER	INER
Hidroxicloroquina	200	INER	SANOFI
Placebo	200	INER	SANOFI
Electrocardiograma	400	INER	INER

Instituto Nacional de Enfermedades Respiratorias Comité de Investigación 25 MAR. 2020 APROBADO INER

Instituto Nacional de Enfermedades Respiratorias Comité de Ética en Investigación 25 MAR. 2020 APROBADO INER

ÚNICAMENTE PARA PROTOCOLOS FINANCIADOS POR LA INDUSTRIA FARMACÉUTICA
BENEFICIOS PARA LA INSTITUCIÓN

Explicar los beneficios: económicos para pacientes, infraestructura u otros, que tendrá el Instituto Nacional de Enfermedades Respiratorias Ismael Cosío Villegas, al participar en el presente protocolo de investigación.

2 5 [illegible]
2 5 [illegible] 20[illegible]
INER
