# Supplementary material for: Hydroxychloroquine for prophylaxis of COVID-19 in health workers: A randomized clinical trial
Source: PLoS One. 2022 Feb 9;17(2):e0261980. doi: 10.1371/journal.pone.0261980 (PMC8827445; doi:10.1371/journal.pone.0261980)
Supplement: S2 File — (PDF) [file pone.0261980.s005.pdf]

**Title of the Project**

Hydroxychloroquine for prophylaxis of symptomatic COVID-19 in healthcare workers: randomized clinical trial.

**Responsible Researcher and Position**

Dr. Jorge Rojas Serrano – Specialist attached to the Intersticial Diseases Unit

**Participant Researchers and Positions**

Dr. Cristóbal Guadarrama – Chief of the Respiratory Urgencies Unit

Dr. Carmen Margarita Hernández Cárdenas – Chief of Critical Areas

Dr. Luis Felipe Jurado Camacho – Specialist attached to the Respiratory Intensive Care Unit

Dr. José Rogelio Pérez Padilla – Chief of the Department of Research in Tobacco and COPD

Dr. Ireri Isadora Thirion Romero – Specialist attached to the Department of Research in Tobacco and COPD

Dr. Sebastián Rodríguez Llamazares – Specialist attached to the Department of Research in Tobacco and COPD

Dr. Alejandra Ramírez Venegas – Specialist attached to the Department of Research in Tobacco and COPD.

**Beginning date:** March 23, 2020

**Ending date:** March 22, 2021

**Schedule in months**

| | 1 | 2 | 3 | 4 | 5 | 6 | 7 | 8 | 9 | 10 | 11 | 12 | 13 | 14 | 15 | 16 | 17 | 18 | 19 | 20 | 21 | 22 | 23 | 24 |
|---|---|---|---|---|---|---|---|---|---|---|---|---|---|---|---|---|---|---|---|---|---|---|---|---|
| Execution | x | x | x | x | x | | | | | | | | | | | | | | | | | | | |
| Analysis | | x | x | x | | | | | | | | | | | | | | | | | | | | |
| Publication preparation | | | x | x | x | | | | | | | | | | | | | | | | | | | |

**Responsible researcher's last 5 publications and/or projects**

Del Angel-Pablo AD, Buendía-Roldán I, Mejía M, Pérez-Rubio G, Nava-Quiroz KJ, Rojas-Serrano J, Falfán-Valencia R. Anti-HLA Class II Antibodies Correlate with C-Reactive Protein Levels in Patients with Rheumatoid Arthritis Associated with Interstitial Lung Disease. Cells. 2020 Mar 11;9(3):691. doi: 10.3390/cells9030691. PMID: 32168865; PMCID: PMC7140697.

Mateos-Toledo H, Mejía-Ávila M, Rodríguez-Barreto Ó, Mejía-Hurtado JG, Rojas-Serrano J, Estrada A, Castillo-Pedroza J, Castillo-Castillo K, Gaxiola M, Buendía-Roldan I, Selman M. An Open-label Study With Pirfenidone on Chronic Hypersensitivity Pneumonitis. Arch Bronconeumol (Engl Ed). 2020 Mar;56(3):163-169. English, Spanish. doi: 10.1016/j.arbres.2019.08.019. Epub 2019 Nov 26. PMID: 31784348.

Cavagna L, Trallero-Araguás E, Meloni F, Cavazzana I, Rojas-Serrano J, Feist E, Zanframundo G, Morandi V, Meyer A, Pereira da Silva JA, Matos Costa CJ, Molberg O, Andersson H, Codullo V, Mosca M, Barsotti S, Neri R, Scirè C, Govoni M, Furini F, Lopez-Longo FJ, Martinez-Barrio J, Schneider U, Lorenz HM, Doria A, Ghirardello A, Ortego-Centeno N, Confalonieri M, Tomietto P, Pipitone N, Rodriguez Cambron AB, Blázquez Cañamero MÁ, Voll RE, Wendel S, Scarpato S, Maurier F, Limonta M, Colombelli P, Giannini M, Geny B, Arrigoni E, Bravi E, Migliorini P, Mathieu A, Piga M, Drott U, Delbrueck C, Bauhammer J, Cagnotto G, Vancheri C, Sambataro G, De Langhe E, Sainaghi PP, Monti C, Gigli Berzolari F, Romano M, Bonella F, Specker C, Schwarting A, Villa Blanco I, Selmi C, Ceribelli A, Nuno L, Mera-Varela A, Perez Gomez N, Fusaro E, Parisi S, Sinigaglia L, Del Papa N, Benucci M, Cimmino MA, Riccieri V, Conti F, Sebastiani GD, Iuliano A, Emmi G, Cammelli

D, Sebastiani M, Manfredi A, Bachiller-Corral J, Sifuentes Giraldo WA, Paolazzi G, Saketkoo LA, Giorgi R, Salaffi F, Cifrian J, Caporali R, Locatelli F, Marchioni E, Pesci A, Dei G, Pozzi MR, Claudia L, Distler J, Knitza J, Schett G, Iannone F, Fornaro M, Franceschini F, Quartuccio L, Gerli R, Bartoloni E, Bellando Randone S, Zampogna G, Gonzalez Perez MI, Mejia M, Vicente E, Triantafyllias K, Lopez-Mejias R, Matucci-Cerinic M, Selva-O'Callaghan A, Castañeda S, Montecucco C, Gonzalez-Gay MA. Influence of Antisynthetase Antibodies Specificities on Antisynthetase Syndrome Clinical Spectrum Time Course. J Clin Med. 2019 Nov 18;8(11):2013. doi: 10.3390/jcm8112013. PMID: 31752231; PMCID: PMC6912490.

Guisado-Vasco P, Silva M, Duarte-Millán MA, Sambataro G, Bertolazzi C, Pavone M, Martín-Garrido I, Martín-Segarra O, Luque-Pinilla JM, Santilli D, Sambataro D, Torrisi SE, Vancheri A, Gutiérrez M, Mejia M, Palmucci S, Mozzani F, Rojas-Serrano J, Vanchieri C, Sverzellati N, Ariani A. Quantitative assessment of interstitial lung disease in Sjögren's syndrome. PLoS One. 2019 Nov 8;14(11):e0224772. doi: 10.1371/journal.pone.0224772. PMID: 31703067; PMCID: PMC6839858.

Ramos-Martínez E, Rojas-Serrano J, García-Hernández O, García-Vázquez FJ, Andrade WA, Avila G, Salinas-Pasquier L, López-Vancell MR. The immune response to Hymenolepis nana in mice decreases tumorigenesis induced by 7,12 dimethylbenz-anthracene. Cytokine. 2019 Nov;123:154743. doi: 10.1016/j.cyto.2019.154743. Epub 2019 Jun 27. PMID: 31255915.

**Title of the Project**

Hydroxychloroquine for prophylaxis of symptomatic COVID-19 in healthcare workers: randomized clinical trial.

**Definition of the Problem**

Healthcare personnel have a high risk of COVID-19 infection (1). In China, until the beginning of March, more than 3300 COVID-19 infections in medical staff have been reported, and in some hospitals such as Clinic Barcelona 23% of physicians have contracted COVID-19. In Italy, the estimated incidence of COVID-19 is similar, with 20% of medical staff infected (1). Another severe problem is that healthcare workers may take the COVID-19 infection to their homes, having transmission to their family members been reported; besides, personnel might be an in-hospital vector, spreading the infection to patients being treated for other causes, or to the rest of the medical team.

Disease in healthcare personnel is an extremely severe problem in health emergencies, since it limits capacity of attention, weakens the workforce and diminishes motivation. In fact, in China 40,000 physicians were mobilized from other regions to support Wuhan and its province in the initial outbreak. Moreover, in Italy, news of hiring physicians from other countries like Cuba, China and Venezuela to support medical care of affected patients spread, since health systems were overwhelmed.

**Research Question**

¿Does prophylactic treatment with hydroxychloroquine 200 mg/day for 60 days significantly reduce the incidence of symptomatic COVID-19 infections in healthcare workers in direct contact with infected patients compared with placebo?

**Background**

The outbreak of respiratory infections due to coronavirus 2019 (COVID-19) began in China in December 2019, in the city of Wuhan, Hubei province (1 – 5). From this city, the outbreak has extended to other Chinese provinces and other countries (6). Until February 16, 2020, 28 countries had recognized confirmed cases, with 70,548 infections and 1,770 deaths in China and 413 infections in Japan (6). As other coronavirus, like SARS and MERS, the original human infection probably came from contact with animals, but effective transmission between humans has been proved, with a growth rate higher than 1 estimated at 2.6 (7) and a rapid duplication of cases that guarantees not only the continuation of the epidemics but its expansion, as has been observed so far.

COVID-19 has a wide range of clinical presentations, from asymptomatic infections to fatal disease with severe lung damage, respiratory failure and multiorganic failure. Severe damage has been associated, as in severe influenza, to a systemic

inflammatory reaction or cytokine storm, that may be attenuated by the immunomodulator effect of chloroquine or hydroxychloroquine. The fatality rate of COVID-19 has been estimated around 2%, but in severe cases with respiratory failure or need of intensive care it is considerably higher, between 10 – 15%, and could easily go up to 20 – 30% depending on the criteria of admission to an intensive respiratory care unit.

Until now there is no effective treatment for COVID-19 available, but it was recently reported that Remdesivir and chloroquine are effective in vitro. This one, in concentrations that can be obtained in vivo (8). Chloroquine and hydroxychloroquine (HCQ) are originally antimalarial drugs, almost identical chemically, that have been in regular use for decades, with a good security profile and accessible cost. The main toxicity of HCQ, investigational drug in this trial; is retinopathy, being its main associated risk factors doses higher than 5 mg/kg weight/day, lengthy use (longer that 10 years) and history of kidney failure, as well as use of tamoxifen. Other adverse effects reported with HCQ, happening scarcely, are alterations of heart conduction, cardiomyopathies, neurological symptoms such as nystagmus, headache, insomnia and nightmares, among others.

The antiviral effect of HCQ has been attributed to the increase in endosomal pH, being endosomes required for viral-cellular fusion, and to interference with glycosylation of the coronavirus (SARS-CoV-2) cellular receptors (9-11). Besides its antiviral effect HCQ also has immunomodulator effects, which are used for treatment of diseases like rheumatoid arthritis and systemic erythematous lupus. This effect may enhance the antiviral effect and diminish the systemic impact of severe infection. In case of being effective as prophylaxis, it could a very useful drug to lower the risk of COVID-19 in healthcare workers.

Recently a letter was published (12), describing how in February 17 the State Council of China had given a press announcement indicating that chloroquine had shown marked efficacy and acceptable security for the treatment of COVID-19 pneumonia in multicentric trials with more than 100 subjects in 10 hospitals. On the other hand, reports of therapeutic success using HCQ in combination with azithromycin have come out, which even being preliminary support the thesis of the antiviral effect of HCQ and its possible use as prophylaxis in subjects at risk of COVID-19. Based on the above, we propose this double blind randomized clinical trial, with two parallel groups, of treatment with HCQ against placebo, to evaluate the efficacy and security of HCQ in healthcare workers with risk of COVID-19.

### Justification

There is a lack of an approved pharmacologic treatment for chemoprophylaxis of COVID-19 and based on the in vitro effectivity of HCQ against the SARS-CoV-2 virus, its security profile and low cost we propose a randomized double blind controlled trial to evaluate the efficacy and security of HCQ to prevent symptomatic and severe infections in the healthcare workers highly exposed to COVID-19. The proposed dosage for prophylaxis is 200 mg/day. This is lower than 5 mg/kg/day in subjects who weight more than 50 kg, which makes the risk of retinal toxicity minimal, since subjects who weigh less than 50 kg will be excluded. In case of proving that the incidence of symptomatic and severe COVID-19 in health workers diminish significantly, this prophylaxis strategy may be one of the best tools to upkeep the security of the healthcare personnel exposed to COVID-19.

### Hypothesis

Alternate hypothesis: Prophylactic treatment with hydroxychloroquine 200 mg/day for 60 days reduces the incidence of symptomatic infections due to SARS-CoV-2 in more than 10% in healthcare workers in contact with infected patients.

Null hypothesis: Prophylactic treatment with hydroxychloroquine 200 mg/day for 60 days doesn't reduce the incidence of symptomatic infections due to SARS-CoV-2 in more than 10% in healthcare workers in contact with infected patients.

### General objectives

To evaluate the efficacy of prophylaxis with HCQ at a 200 mg/day dosage, compared against placebo, to diminish the incidence of symptomatic COVID-19 in healthcare workers with high COVID-19 exposure.

To evaluate the security of prophylaxis with HCQ at a 200 mg/day dosage, compared against placebo, to diminish the incidence of symptomatic COVID-19 in healthcare workers with high COVID-19 exposure.

**Specific objectives**

To compare the risk of symptomatic infection with COVID-19 in subjects treated with prophylaxis with hydroxychloroquine against the risk of symptomatic infection with COVID-19 in subjects treated with placebo.

To estimate the risk of presenting the composite outcome (admission to intensive respiratory care unit, need of mechanical invasive /non-invasive ventilation and death) among subjects with high exposure to COVID-19, treated with prophylaxis with HCQ against those treated with placebo.

To estimate the risk of adverse effects in healthcare workers treated with prophylaxis with hydroxychloroquine against the risk of adverse effects in subjects treated with placebo.

**Experimental design**

Clinical research.

Experimental, longitudinal, prospective study.

This study will obtain knowledge for the prevention of COVID-19.

The research line from which this project derivates is the program of study about influenza and other respiratory viruses.

**Material and Methods**

<u>Place of study</u>

Instituto Nacional de Enfermedades Respiratorias “Ismael Cosío Villegas” (National Institute of Respiratory Diseases, INER); areas of the Urgencies, Critical Medicine and Hospitalization Units.

<u>Description of population</u>

Healthcare workers from INER, in contact with the attention of COVID-19 patients, including medical personnel, nursing practitioners, and paramedics such as stretcher handlers and cleaning staff. The INER workers included will have taken training courses on the use of biosecurity protection, with the goal of lowering the risk of COVID-19 infection.

<u>Procedures of the study</u>

Phase III double blind controlled randomized trial, with two parallel groups.

*Randomization*

Healthcare workers exposed to patients with COVID-19 diagnosis will be distributed randomly in a stratified way. The strata for randomization will be the following:

a) Group A: subjects with high-risk exposition to COVID-19; defined as the personnel who perform high risk invasive procedures (bronchoscopy, intubation, extubation and/or respiratory sample taking), care of critical or under mechanical ventilation patient.
b) Group B: subjects with low to moderate risk exposition to COVDI-19; defined as the personnel who attend patients in triage or outpatient areas, or whose participation in the Unit corresponds to cleaning or administration labour.

The randomization distribution will be performed with the permutated block method with random allocation, in blocks of four pairs.

*Blind description*

Subject, researcher, treating physician, outcome monitor, results analysis.

*Main outcome*

Symptomatic infection (dry cough with or without dyspnea, with or without fever) due to COVID-19 proved via PCR-TR according to the OMS protocol.

*Secondary outcomes*

1. Admission to the Intensive Respiratory Care Unit , need of invasive/non-invasive mechanical ventilation, death secondary to COVID-19, proved via PCR-TR according to the OMS protocol.
2. Incidence and severity of adverse events
   Adverse event: all medical events in the subjects included in the trial. The adverse event doesn't necessarily need to have a causal relation with the studied treatment. Examples of adverse events include abnormal symptoms and signs, hypersensitivity reactions or alterations in laboratory samples.
   Severe adverse event: defined as an event that results in the subject's death, in an event that threatens life, that requires hospital admission or causes incapacity.
   Due to the known effects of HCQ, special attention will be given to the incidence of the following adverse events:
   a) Cutaneous reaction, generally when used along other drugs.
   b) Medullar suppression, which must be watched over monthly through a control blood count.
   c) Severe hypoglycemia, in which case the treatment must be suspended.
   d) Proximal myopathy with proximal weakness or muscular atrophy.
   e) Increase of suicidal risk according to the C-SSRS scale.

   Adverse reactions are defined as the answer to a compound that is harmful and involuntary, that occurs at doses that normally are administrated to human beings. The described adverse reactions are:

   1- 10% ophthalmologic, dose dependent.
   2- Non-defined frequency: dermatologic, endocrine, metabolic, gastrointestinal, hematologic, hepatic, central nervous system, immunologic.

   Gradation of adverse reactions

   Grade 1: mild adverse event: refers to the effects that may happen due to the compound that are temporal and don't cause permanent harm or incapacity.

   Grade 2: moderate adverse event; refers to the effects that may happen due to the compound that are temporal but require support from other therapeutics to be corrected and don't cause permanent harm or incapacity.

   Grade 3: severe adverse event; refers to the effects that may happen due to the compound that might be temporal or permanent, without risk of mortality or incapacity.

   Grade 4: adverse advent with risk of death or incapacity.

   Evaluated through the following study:

   Electrocardiogram: QT interval prolongation.

   Blood count: bone medulla suppression, defined with the following values: neutrophiles < 500/microliters, platelets < 20,000/microliter, reticulocites < 20,000 / microliter.

Hepatic failure: INR> 1.4, ALT> 33 units/liter, AST > 55 units/liter, total bilirubin > 4 mg/dl, platelets < 150,000/microliter.

Due to the nature of the study, having a captive population that comes to the INER at least five days a week, it is possible to estimate the number of days to the outcome. All participants will be instructed to notify the researchers in case of presenting cough, dyspnea or any other new sign or symptom. Weekly researchers will ask all included subjects about the symptoms associated with COVID-19, as well as any possible adverse events.

If any study subject presents adverse reactions, the treatment will begin immediately, and it will be notified at the same time. The initial treatment will be administered at the INER, support from other institutions will be requested, if necessary, without cost for the subject.

*Experimental group*

Subjects in this group will receive HCQ orally, 200 mg/day for 60 days. According to the result of the interim analysis at 4 and 8 weeks it will be decided whether to remove the blind and giving the prophylaxis to both groups or not. According to the results of the trial, an amend may be made to continue with an open label study, studying prophylaxis with HCQ.

*Control group*

Similar placebo for 60 days.

*Speed of recruitment*

All assigned workers will be recruited as the arrive to their work shifts.

*Start of recruitment*

Immediately after the revision process and, if applicable, approval of the protocol.

*Ending of recruitment*

*Tests to perform*

- Minimum tests at recruitment (before randomization) and at 30 and 60 days:
    - Blood count
    - Serum electrolytes
    - Blood chemistry (3 elements): to quantify the effects on blood sugar and rule out type 2 diabetes mellitus at baseline.
    - Hepatic profile
    - Electrocardiogram: to evaluate the incidence of heart conduction alterations.
- 10-respiratory virus and SARS-CoV-2 panel, only in case of symptoms.

At 30 and 60 days: surveillance of adverse events.

<u>Necessary number of subjects</u>

*Significance level*

5% alpha at two tails.

*Power*

(1-beta, possibility of detection): 90%

*Final calculation*

According to the proposed statistical analysis, the difference in time to outcome will be performed with a log-rank test, using the following formulas:

$$n = \frac{d}{2 - S1(\infty) - S2(\infty)} \text{ y } d = (Z\alpha(2) - Z1 - \beta)^2$$

Considering a difference of 10% of higher survival rates free of COVID-19 and considering a reported survival rate free of COVID-19 of 76%, a sample size of 159 per group is obtained. Considering the losses, it is decided that 200 subjects will be included per group, for a total of 400 subjects.

<u>Inclusion and exclusion criteria</u>

*Inclusion criteria*

- At least 18 years of age
- Being a healthcare worker exposed to respiratory disease due to COVID-19: medical personnel, chemists, nurse practitioners, cleaning staff, stretch workers, transportation workers, administrative personnel and respiratory therapy; with training in biosecurity measures and proper use of respiratory protection equipment to lower the risk of COVID-19.
- Absence of respiratory symptoms at the randomization and start of treatment/placebo.
- Informed consent, including randomization to any group.

*Exclusion criteria*

- Known hypersensitivity to HCQ, evidenced by anaphylaxis.
- Current treatment with HCQ or chloroquine.
- Weight less than 50 kg at the basal evaluation.
- Women with last menstrual date a month before recruitment without a pregnancy test.
- Women with positive pregnancy test.
- Lactating women.
- History of chronic hepatic disease (Child-Pugh B or C)
- History of chronic kidney disease (GFR lower or equal to 30)
- History of treatment with tamoxifen.
- History of retinopathy of any cause, and/or loss of central vision, loss of nocturnal visual acuity, blurred vision and alteration of color vision.

*Elimination criteria*

- Decision from the participant or their legal representative to withdraw from the study

<u>Statistical analysis</u>

Categorical variables will be described with frequencies and percentages, numerical variables with mean and standard deviation or median and interquartile range according to their distribution. The time to the outcome (COVID-19) and to the compound outcome will be estimated, in both the experimental and the placebo group. The survival function will be estimated with the Kaplan – Meier method and the log rank hypothesis test will be used to contrast the time to outcome in each study group. The hazard ratio and its 95% confidence interval will be used as measure of association strength to evaluate the effect of the prophylactic HCQ over the main and secondary outcomes of the study. The effect of prophylaxis on both proposed

study strata will be estimated with the log rank test, and the interaction prophylaxis – strata with be evaluated with Cox regression. Due to the urgency of knowing the study results, the following interim analysis are proposed: at the moment that the 50% of the sample has had a follow-up of four weeks and at the moment that 50% of the sample has had 8 weeks of follow-up. The subjects that do not present the outcome will be censured in their follow-up at three months from the random allocation and start of the intervention (HCQ/placebo). We propose a multivariate Cox analysis to evaluate the risks associated with COVID-19 and risk of the composite outcome. All analysis will be two-tailed, the analysis strategy is intention to treat, and all randomized subjects will be included in the analysis.

*Data and security monitoring committee (DSMC)*

A DSMC will take part in the study, being a multidisciplinary advisor group formed by a biostatistics expert, a clinical researcher, an ethics expert and a clinical involved with influenza and coronavirus, none of whom will be involved in the clinical trial and are therefore independent, who, at proposed times, will have access to the data and randomization list so that they can evaluate the data including security and outcomes.

Clinical researcher: Gustavo Lugo, clinical pharmacologist, anesthetist and intensivist who will act as coordinator.

Statistician: MSc María del Rosario Fernández Plata, Chief of the Epidemiologic Department, INER.

Ethics: Dr. Patricio Santillán Doherty, Medical Director, INER.

Clinician: Dr. Arturo Martínez, specialist in Infectious Diseases, expert in influenza-like infections and now in COVID-19.

Their goal will be to valuate advantages and disadvantages of continuing the trial having recruited half the proposed sample size, above all if it is considered ethical ton continue recruitment, given the outcomes found at this time. At this moment there are not controlled studies to compare this with, but they may be made public during the study.

An interim analysis is planned when half the sample is completed, so that a noticeable difference may be identified in the main outcome, which may show an advantage in effectiveness, that for its magnitude forces the trial to stop. Adverse events or toxicity will also be watched for. The final statistical analysis will be made considering this intermediate analysis. }

**Ethical considerations**

This trial is considered at risk higher than minimal, according to what is established in article 1 of the Regalement of the General Health Law in the Subject of Health Research.

All subjects will be included only after reading and signing of the informed consent form.

The risks that the participant subjects will be exposed to are the following:

1. Due to the taking of the blood sample at baseline, 30 and 60 days, the associated risks are venipuncture pain, infection and hematoma.
2. Due to the electrocardiogram, the associated risks are ecchymosys secondary to the electrodes.
3. Due to the swab sample, either nasal or pharyngeal, to use in the PCR-TR to detect SARS-CoV-2 the associated risks are pain, mild erosion of the pharyngeal mucosa with or without slight bleeding.

The expectative of the effects of treatment with HCQ, in case of being assigned to the treatment group, is that there is a lower risk of presenting symptomatic COVID-19, but with a higher risk of prophylaxis adverse events .

The expectative of effects due to placebo treatment, in case of being assigned to that group, is presenting symptomatic COVID-19 infection, at the same risk as if the subject hadn't been recruited in the study, at the cost of the nuisances mentioned in sample taking.

In case the trial shows a clear benefit with HCQ prophylaxis, with lower COVID-19 incidence, an amend will be proposed to remove the blind, either at week 4 or week 8, or at the end of the original 3 months proposed of follow-up.

**Commentary and considerations of the researchers, training of new human resources during the project**

This protocol is part of the doctoral thesis to obtain the degree of Phylosophy Doctor of student Luis Felipe Jurado Camacho.

**Bibliography**

UTILIZAR LAS HOJAS NECESARIAS


1. Joob B, Wiwanitkit V. COVID-19 in medical personnel: observation from Thailand. *The Journal of hospital infection* 2020.

2. Chan JF, Yuan S, Kok KH, To KK, Chu H, Yang J, Xing F, Liu J, Yip CC, Poon RW, Tsoi HW, Lo SK, Chan KH, Poon VK, Chan WM, Ip JD, Cai JP, Cheng VC, Chen H, Hui CK, Yuen KY. A familial cluster of pneumonia associated with the 2019 novel coronavirus indicating person-to-person transmission: a study of a family cluster. *Lancet* 2020.

3. Chen N, Zhou M, Dong X, Qu J, Gong F, Han Y, Qiu Y, Wang J, Liu Y, Wei Y, Xia J, Yu T, Zhang X, Zhang L. Epidemiological and clinical characteristics of 99 cases of 2019 novel coronavirus pneumonia in Wuhan, China: a descriptive study. *Lancet* 2020.

4. Holshue ML, DeBolt C, Lindquist S, Lofy KH, Wiesman J, Bruce H, Spitters C, Ericson K, Wilkerson S, Tural A, Diaz G, Cohn A, Fox L, Patel A, Gerber SI, Kim L, Tong S, Lu X, Lindstrom S, Pallansch MA, Weldon WC, Biggs HM, Uyeki TM, Pillai SK, Washington State -nCo VCIT. First Case of 2019 Novel Coronavirus in the United States. *The New England journal of medicine* 2020.

5. Zhou P, Yang XL, Wang XG, Hu B, Zhang L, Zhang W, Si HR, Zhu Y, Li B, Huang CL, Chen HD, Chen J, Luo Y, Guo H, Jiang RD, Liu MQ, Chen Y, Shen XR, Wang X, Zheng XS, Zhao K, Chen QJ, Deng F, Liu LL, Yan B, Zhan FX, Wang YY, Xiao GF, Shi ZL. A pneumonia outbreak associated with a new coronavirus of probable bat origin. *Nature* 2020.

6. Zhu N, Zhang D, Wang W, Li X, Yang B, Song J, Zhao X, Huang B, Shi W, Lu R, Niu P, Zhan F, Ma X, Wang D, Xu W, Wu G, Gao GF, Tan W, China Novel Coronavirus I, Research T. A Novel Coronavirus from Patients with Pneumonia in China, 2019. *The New England journal of medicine* 2020.

7. Centers of Disease Control. 2019 Novel Coronavirus. 2020 [cited 2020 February 7]; Available from: https://www.cdc.gov/coronavirus/2019-ncov/locations-confirmed-cases.html

8. Zhao S, Musa SS, Lin Q, Ran J, Yang G, Wang W, Lou Y, Yang L, Gao D, He D, Wang MH. Estimating the Unreported Number of Novel Coronavirus (2019-nCoV) Cases in China in the First Half of January 2020: A Data-Driven Modelling Analysis of the Early Outbreak. *Journal of clinical medicine* 2020: 9(2).

9. Wang M, Cao R, Zhang L, Yang X, Liu J, Xu M, Shi Z, Hu Z, Zhong W, Xiao G. Remdesivir and chloroquine effectively inhibit the recently emerged novel coronavirus (2019-nCoV) in vitro. *Cell research* 2020.

10. Savarino A. Use of chloroquine in viral diseases. *The Lancet Infectious diseases* 2011: 11(9): 653-654.

11. Savarino A, Boelaert JR, Cassone A, Majori G, Cauda R. Effects of chloroquine on viral infections: an old drug against today's diseases? *The Lancet Infectious diseases* 2003: 3(11): 722-727.

12. Yan Y, Zou Z, Sun Y, Li X, Xu KF, Wei Y, Jin N, Jiang C. Anti-malaria drug chloroquine is highly effective in treating avian influenza A H5N1 virus infection in an animal model. *Cell research* 2013: 23(2): 300-302.

13. Gao J, Tian Z, Yang X. Breakthrough: Chloroquine phosphate has shown apparent efficacy in treatment of COVID-19 associated pneumonia in clinical studies. *Bioscience trends* 2020.

**Specification of human and technical resources required for the study.**

Specify those that require authorization from the Auxiliary Diagnostical and Paramedical Services Direction

| Resource | Required number | Origin or place to be used | Source of financing |
|---|---|---|---|
| Main researcher | 1 | INER | INER |
| Sub researchers | 8 | INER | INER |
| Study coordinator | 1 | INER | CONACYT |
| Executors | 3 | INER | CONACYT |
| Laboratory kits | 400 | INER | INER |
| Hydroxychloroquine | 200 | INER | SANOFI |
| Placebo | 200 | INER | SANOFI |
| Electrocardiogram | 400 | INER | INER |
